# A Mechanistic and Pathophysiological Approach for Stroke Associated with Drugs of Abuse

**DOI:** 10.3390/jcm8091295

**Published:** 2019-08-23

**Authors:** Aristides Tsatsakis, Anca Oana Docea, Daniela Calina, Konstantinos Tsarouhas, Laura-Maria Zamfira, Radu Mitrut, Javad Sharifi-Rad, Leda Kovatsi, Vasileios Siokas, Efthimios Dardiotis, Nikolaos Drakoulis, George Lazopoulos, Christina Tsitsimpikou, Panayiotis Mitsias, Monica Neagu

**Affiliations:** 1 Center of Toxicology Science & Research, Medical School, University of Crete, 71003 Heraklion, Crete, Greece; 2 Department of Toxicology, University of Medicine and Pharmacy of Craiova, 200349 Craiova, Romania; 3 Department of Clinical Pharmacy, University of Medicine and Pharmacy of Craiova, 200349 Craiova, Romania; 4 Department of Cardiology, University Hospital of Larissa, 41221 Larissa, Greece; 5 Department of Pathology, University of Medicine and Pharmacy of Craiova, 200349 Craiova, Romania; 6 Department of Cardiology, University and Emergency Hospital, 050098 Bucharest, Romania; 7 Zabol Medicinal Plants Research Center, Zabol University of Medical Sciences, Zabol 61615-585, Iran; 8 Laboratory of Forensic Medicine and Toxicology, School of Medicine, Aristotle University of Thessaloniki, 54248 Thessaloniki, Greece; 9 Department of Neurology, Stroke Unit, University of Thessaly, University Hospital of Larissa, 41221 Larissa, Greece; 10 Research Group of Clinical Pharmacology and Pharmacogenomics, Faculty of Pharmacy, School of Health Sciences, National and Kapodistrian University of Athens, 15771 Athens, Greece; 11 Department of Cardiothoracic Surgery, University General Hospital of Heraklion, University of Crete, Medical School, 71003 Heraklion, Crete, Greece; 12 Department of Hazardous Substances, Mixtures and Articles, General Chemical State Laboratory of Greece, 10431 Athens, Greece; 13 Department of Neurology, School of Medicine, University of Crete, 71003 Heraklion, Greece; 14 Comprehensive Stroke Center and Department of Neurology, Henry Ford Hospital, Detroit, MI 48202, USA; 15 Department of Immunology, Victor Babes National Institute of Pathology, 050096 Bucharest, Romania; 16 Department of Pathology, Colentina Clinical Hospital, 021183 Bucharest, Romania

**Keywords:** stroke, amphetamines, cocaine, cannabis, morphine, heroin, synthetic cannabinoids, anabolic androgenic steroids

## Abstract

Drugs of abuse are associated with stroke, especially in young individuals. The major classes of drugs linked to stroke are cocaine, amphetamines, heroin, morphine, cannabis, and new synthetic cannabinoids, along with androgenic anabolic steroids (AASs). Both ischemic and hemorrhagic stroke have been reported due to drug abuse. Several common mechanisms have been identified, such as arrhythmias and cardioembolism, hypoxia, vascular toxicity, vascular spasm and effects on the thrombotic mechanism, as causes for ischemic stroke. For hemorrhagic stroke, acute hypertension, aneurysm formation/rupture and angiitis-like changes have been implicated. In AAS abuse, the effect of blood pressure is rather substance specific, whereas increased erythropoiesis usually leads to thromboembolism. Transient vasospasm, caused by synthetic cannabinoids, could lead to ischemic stroke. Opiates often cause infective endocarditis, resulting in ischemic stroke and hypereosinophilia accompanied by pyogenic arthritis, provoking hemorrhagic stroke. Genetic variants are linked to increased risk for stroke in cocaine abuse. The fact that case reports on cannabis-induced stroke usually refer to the young population is very alarming.

## 1. Introduction

### 1.1. Stroke Definitions

According to the World Health Organization, a stroke is defined as ‘a clinical syndrome consisting of rapidly developing clinical signs of focal (or global in case of coma) disturbance of cerebral function lasting more than 24 h or leading to death with no apparent cause other than a vascular origin’. On the other hand, a transient ischemic attack (TIA) presents the signs and symptoms of a stroke, but without tissue damage and the symptoms usually resolve within 24 h [1,2]. A stroke can be defined as a rupture or blockage of an artery of the brain, which results in bleeding into the brain parenchyma or in decreased blood supply and ischemic damage to specific brain areas respectively [3].

### 1.2. Epidemiology of Illicit Drugs of Abuse Use and Stroke

The use of psychoactive substances has been known for thousands of years: From the ingestion of plant derivatives, such as the mushroom *Psilocybe hispanica* used in religious rituals performed 6000 years ago, to the abuse of synthetic drugs, such as heroin that was first synthesized in 1874 by C. R. Alder Wright, an English chemist working at St. Mary’s Hospital Medical School in London. Nowadays, substance abuse constitutes a major social and medical problem. According to the World Drug Report 2017, issued by the United Nations Office on Drugs and Crime, the number of estimated drug users worldwide has increased by 23% in 11 years, reaching 255 million individuals in 2015. At the same time, drug users with various health disorders, such as lung or heart disease, mental health diseases, infectious diseases, stroke and cancer, reached 29.5 million in 2015, with an increase of 13.5% compared to 2006. The number of deaths attributed to drug abuse has also significantly increased. Out of the total registered deaths due to drug abuse, 67.5% are attributed to amphetamine use, 49.7% to cocaine, 29.6% to opioids and the remaining 23% to other drugs [4].

Stroke is the second leading cause of death in the world, responsible for 5.7 million deaths every year, which is expected to reach approximately 7.8 million by 2030 [5,6,7,8]. Moreover, stroke is the leading cause of major disability. A timely diagnosis by computed tomography (CT) and, depending on the circumstances, by CT angiography and CT perfusion is necessary to assure effective management [3,7].

### 1.3. Classic Concept of Stroke Pathophysiology

A stroke occurs when blood circulation of the brain is disturbed. There are two types of strokes: Ischemic stroke/transient ischemic attack (TIA) and hemorrhagic stroke. Brain tissue destruction is caused by different mechanisms with multifactorial character in the two types of strokes.

Ischemic stroke represents the loss of brain function caused by a decreased blood flow and consequently reduced oxygen supply to the affected brain tissue [9].

The knowledge of the latest physiopathological mechanisms in ischemic stroke is important for the development of new pharmacotherapies. Recent experimental studies in mice with transient middle cerebral artery occlusion (tMCAO) have shown the involvement of the Von Willebrandt factor (vWF) which interacts with and binds to the GPI platelet glycoprotein and the collagen receptor GP VI [10]. This vWF–GPIb axis combined with activated coagulation factor XII triggers the thrombo-inflammatory cascade in acute ischemic stroke [10,11]. In this thrombo-inflammatory process, platelets interact with T cells, which aggravate ischemia-reperfusion injury after recanalization [10,11]. However, targeting stroke-related neuroinflammation with anti-inflammatory drugs may be used with caution in order to detect any potential adverse effects to be avoided [11].

Numerous other pathophysiological studies performed on patients with ischemic stroke demonstrated hemostatic abnormalities such as low serum levels of coagulation factor VII, FVII-activated antithrombin complex, tissue factor and increased serum levels of tissue factor-bearing microparticles (MPs-TF) [12,13].

In hemorrhagic stroke the neuronal injury is supplemented by the compressive effect exerted by the hematoma, the systemic inflammatory response, the neuronal toxicity of the hemoglobin and the effect thrombolysis inside the intracerebral thrombus [14,15].

A key role in controlling stroke mortality lies in controlling the so-called modifiable stroke risk factors [3]. There are several risk factors for stroke including age, gender, hypertension, diabetes mellitus, dyslipidemia, atheromatosis, thrombophilia, atrial fibrillation, sick sinus syndrome, patent foramen ovale or family history of cardiovascular events, hyperhomocysteinemia as well as lifestyle habits, such as low physical activity, obesity, tobacco smoking, poor diet, and alcohol consumption [3,5,6,8,16,17,18]. Controlling blood pressure and blood glucose levels, using statins for elevated blood lipid levels and reducing the use of oral contraceptives, along with lifestyle changes, can drastically reduce the risk for stroke [5].

Drugs of abuse are also associated with stroke, especially in younger individuals. It has been shown that drug users, between 15 and 44 years old, were 6.5 times more likely to have a stroke compared with non-users [19]. The major classes of drugs linked to stroke are cocaine, amphetamines, heroin, morphine, cannabis, and the new synthetic cannabinoids, along with androgenic anabolic steroids, which are widely used both by professional and recreational athletes but also by the general public.

This article aims to review epidemiological evidence related to drug abuse-associated stroke and elucidate the possible underlying mechanisms of stroke induced by different classes of drugs of abuse.

## 2. Stroke Linked to Illicit Drugs of Abuse

In general, drugs of abuse can provoke stroke either by causing direct damage to cerebral vessels or indirectly, by affecting other organs, such as the liver (affecting blood coagulation pathways) or the heart, thus negatively affecting cerebral circulation [20,21]. There are substance-specific mechanisms involved. For example, stimulants such as amphetamines, cocaine and their derivatives are associated with both types of stroke, acute ischemic (cerebral infarcts) and hemorrhagic (intracerebral hemorrhages, subarachnoid hemorrhages), where the involved mechanisms differ [21,22].

The increase in blood pressure, caused by stimulants, could lead to a cerebral vessel rupture or aneurysm rupture and a subsequent hemorrhagic stroke. On the other hand, acute ischemic stroke can be attributed to stimulant-induced cerebral vasoconstriction, which reduces blood flow, promotes platelet aggregation and accelerates atherosclerosis and cardiac disturbances [21].

The pathophysiology of stroke, related to drugs of abuse, will be discussed hereafter separately for each class identified.

### 2.1. Amphetamines and Amphetamine Derivatives

Amphetamines are weak bases, chemically similar to natural neurotransmitters, adrenaline, and dopamine. They are synthetic sympathomimetics, which are used as mental stimulants. Their use has increased significantly, mainly because of the euphoria they induce [23]. Amphetamine derivatives include 3,4-methylenedioxymeth-amphetamine (MDMA), N-ethyl-3,4-methylenedioxyamphetamine (MDEA), 3,4-methylenedioxy-amphetamine (MDA) and methylenedioxymethylpropyl-amphetamine (MDMPA).

#### 2.1.1. Mechanisms of Actions of Amphetamines and Amphetamine Derivatives

All amphetamines are rapidly absorbed when taken orally and even faster when they are smoked, chewed or injected [24]. Tolerance develops to standard and designer amphetamines, leading to the need to increase the dose by the consumer. Classical amphetamines, dextroamphetamine, methamphetamine and methylphenidate produce their primary effects through the release of catecholamines, especially dopamine, in the brain [24,25].

These effects are particularly strong in the brain areas associated with pleasure, especially in the cerebral cortex and limbic system. The effect of this pathway is probably responsible for the amphetamine addiction [24]. Catecholamines are similar to natural body compounds and act as neurotransmitters in the central nervous system [25]. Dopamine, an intermediate derived from epinephrine and norepinephrine biosynthesis is one of these compounds [26]. “Designer amphetamines”, especially Ecstasy, cause the release of catecholamines, dopamine and norepinephrine, in addition to serotonin, a neurotransmitter that produces hallucinogens effects [27].

The main effects of amphetamines are euphoria, increased productivity and motor movements and decreased appetite. In chronic users, amphetamines create tolerance, addiction, and craving [28].

#### 2.1.2. Influence of Amphetamines and Amphetamine Derivatives on Stroke

Amphetamines, which were initially used to increase intellectual performance and weight loss, are associated with both types of stroke [29,30,31].

There is also limited evidence that links a delayed ischemic stroke with amphetamine use, such as the case of a 19-year-old woman who developed right occipital infarction 3 months after methamphetamine use [32]. The mechanism involved in triggering delayed ischemic stroke remains unknown but it seems to be associated with chronic vasculitis [32,33].

Intracranial hemorrhage, following amphetamine abuse, is associated with a transient increase in blood pressure [34]. High blood pressure and vasoconstriction may also occur after consuming the so-called "diet pills" containing the amphetamine-like substance [31,35].

**Figure 1:**
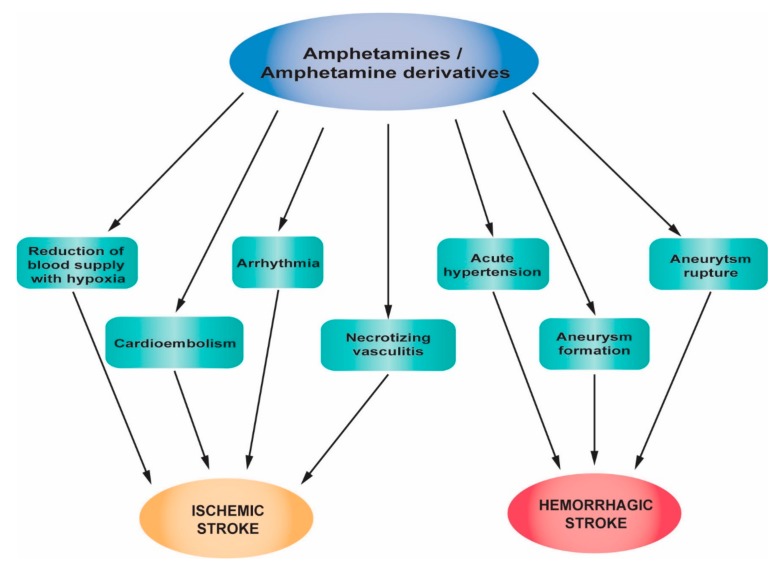
Pathophysiological mechanisms of stroke associated with amphetamines and amphetamine derivative abuse.

An in vivo study on mice revealed that even a single, acute exposure to methamphetamine can induce a biphasic effect in cerebral blood flow: An initial transient increase, followed by a prolonged decrease, 30 min after exposure, that induces vasoconstriction of pial arterioles [36]. Moreover, stroke may be attributed to the direct toxic effect of amphetamines on cerebral vessels, causing necrotizing vasculitis [37]. Many studies report intracranial hemorrhage following the use of amphetamines [38,39]. Figure 1 summarizes the main pathophysiological mechanisms of stroke associated with amphetamines and amphetamine derivative abuse.

#### 2.1.3. Clinical Studies, Case Reports and Epidemiology of Stroke Related to Amphetamines and Amphetamine Derivatives abuse

Amphetamines were first used during World War II by soldiers in order to suppress fatigue. In the 1950s, the legal prescription of amphetamines in the US increased. Worldwide, there are over 35 million people who abuse amphetamines, compared to 15 million cocaine users [28]. The route of administration can be intravenous, oral, intranasal and by inhalation (smoking) [40]. The half-life is between 10 and 30 h and they are metabolized through the liver. Studies have shown that adolescents who use amphetamines have a 5-fold higher risk of stroke than those who do not use these drugs [24,41].

Apart from thrombosis and cerebrovascular pathology, several other side effects of amphetamines and amphetamine derivatives have been reported, including cardiomyopathy and arrhythmias, liver failure, renal failure, suicide, confusion, memory loss, psychosis and premature mortality [40]. The risk of stroke is four times higher in amphetamine users than in nonusers and the hemorrhagic stroke may occur twice as often, as in the case of cocaine users [29]. Although it is less frequent compared to amphetamine-associated hemorrhagic stroke, amphetamine-associated ischemic stroke is also described in the literature (Table 1). De Silva reported the case of a 30-year-old woman who developed acute left middle cerebral artery infarction after acute intake of amphetamine [33]. Christensen et al. reported the case of a 33-year-old Caucasian male addicted to amphetamines who died due to bilateral cerebral infarction [42]. In the past, it was believed that amphetamine derivatives were a safer option compared with other stimulants, because it was thought that intracranial hemorrhages occurred only in combination with other stimulant drugs [39]. However, it was later shown that a clear association exists between intracerebral hemorrhage in young people, without comorbidities, and amphetamine, methamphetamine or their derivative intake [43,44,45]. The most interesting and recent studies that report amphetamine-associated hemorrhagic stroke and amphetamine-associated ischemic stroke are summarized in Table 1. 

### 2.2. Cocaine

Cocaine, also known as benzoylmethylecgonine, is extracted from the leaves of the *Erythroxylum coca* shrub, which usually grows in Peru, Bolivia, and Ecuador [50]. In the past, the leaves of this plant were chewed or sucked in order to decrease hunger or obtain euphoric effect. Its use increased after the 1970s. After 2007, cocaine has become one of the most abused drugs, regularly used by five million Americans [50]. Cocaine has two chemical forms: Cocaine hydrochloride and alkaloidal cocaine [50]. Cocaine hydrochloride is water soluble and is readily absorbed after nasal administration [50]. Alkaloidal cocaine is lipid soluble and is a free base. It is synthesized by mixing cocaine hydrochloride with water and ammonia. Another form is produced by mixing cocaine hydrochloride with sodium bicarbonate, known as ‘crack cocaine’ in street language.

#### 2.2.1. The Mechanism of Action of Cocaine

The main mechanism of action of cocaine is the blockage of noradrenaline reuptake [51]. The side effect is increased norepinephrine release. These effects act synergistically to increase the level of norepinephrine in the nerve endings. Cocaine also causes moderate release and blocking the reuptake of serotonin and dopamine [51]. It is a local anesthetic with effects caused by the blocking of the sodium channels, which determines the inhibition of nerve conduction by decreasing the amplitude of the action potential of the membranes but increasing its duration. Cocaine also blocks the potassium channels and, in some cells, it also blocks the sodium–calcium pump [52]. The drug is soluble in lipids and, therefore, crosses the blood–brain barrier. Cocaine stimulates the central nervous system, especially the limbic system where it potentiates dopaminergic transmission in the basal ventral nuclei, producing the sensation of pleasure, which has led to its widespread use [52]. Cocaine substitutes dopamine, the neurotransmitter involved in mood management [53]. Cocaine use is associated with myocardial infarction, vasoconstriction, chronic uncontrolled hypertension, nervous system stimulation and stroke [53]. Cocaine is associated with vascular toxicity. Various mechanisms are involved, such as hypertension, disturbance of platelet aggregation and homeostasis, effects on cerebral blood flow, and thromboembolism [54].

#### 2.2.2. Influence of Cocaine on Stroke

The risk of stroke is twice as high in cocaine users, compared to age-matched non-users [29,54].

Ischemic stroke related to cocaine is associated with large vessel atherosclerosis, advanced atherosclerosis of intracranial vessels, increased platelet activation and arrhythmias, especially bradyarrhythmias, which can be explained by the ability of cocaine to depress sinus node automaticity and to block the atrioventricular node conduction [53,55].

Although it is well documented that cocaine can cause cerebral ischemia, researchers could not explain the exact mechanism. Cerebral vasospasm is attributed to the sympathomimetic effect of cocaine and the increase in circulating endothelin-1 [56]. Endothelin-1 is a vasoconstrictor protein produced by vascular endothelial cells. When elevated, it leads to nitric oxide decrease and vasoconstriction. In addition, cocaine effects on vasoconstriction are also related to elevated calcium in the vessels [57]. Other causes of stroke, related to acute cocaine use, cervicocephalic or intracranial arterial dissection are additional causes of stroke related to acute cocaine use [53]. A study by You et al. revealed that cocaine can cause a stroke by reducing blood flow to the brain. The researchers visualized exactly what happens in the brain when it is exposed to cocaine. Using quantitative laser-based visualization, it was possible to see exactly how cocaine affects small blood vessels in the brains of mice. Following 30 days of exposure to cocaine (by injection), or even after several injections performed at different time points with short intervals between them, a drastic reduction in blood circulation could be demonstrated. It was shown that in some vessels, cocaine induced micro-ischemia, a state in which blood flow to the brain is not adequate and cerebral hypoxia and ischemic stroke occur. These findings could help physicians to improve neurosurgical techniques and develop more effective methods for treating cocaine users [58].

In a large cohort study on cocaine-related stroke, during a period of 10 years, atherosclerosis of large vessels was found to be the common mechanism of stroke [59]. Cocaine use creates an elevated immune system inflammatory state. Various basal anti-inflammatory markers, like interleukin-10 (IL 10) have been found to be decreased, while pro-inflammatory cytokines (tumor necrosis factor alpha, Interleukin 1β) are increased, thus contributing to vascular disease [60,61].

Acute cocaine use induces acute hypertension, which is implicated in the occurrence of hemorrhagic stroke in users. The implication of cocaine in aneurysm formation and rupture is supported by the high incidence of aneurysmal subarachnoid hemorrhage (SAH) in cocaine users. Only less than half of them have a family history of hypertension [59].

**Figure 2:**
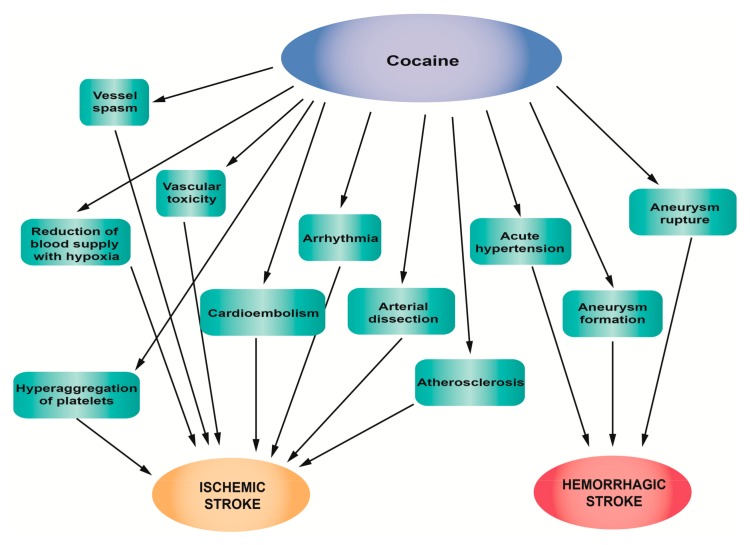
Pathophysiological mechanisms of stroke associated with cocaine abuse.

Figure 2 summarizes the main pathophysiological mechanisms of stroke associated with cocaine abuse.

#### 2.2.3. Clinical Studies, Case Reports and Epidemiology of Stroke Related to Cocaine Abuse

In order to evaluate the net effect of cocaine abuse on stroke risk, co-triggers, predictors, and co-morbidities of stroke (and other vascular diseases) should be taken into account [62]. Factors such as infections with human immunodeficiency virus type 1 (HIV-1) and hepatitis C virus (HCV) are of great importance [63,64]. So far, studies have supported the fact that cocaine use is prevalent in individuals infected with HIV and/or HCV, and vice versa [63,64,65]. Moreover, a few clinical studies suggested that cocaine abuse may increase HIV-1 viral load, and thus increase acquired immune deficiency syndrome (AIDS)-related mortality, even among patients under antiretroviral therapy (ART) [65]. In a study by Lucas et al. (2015), HIV and HCV infection were associated with carotid plaque progression. Furthermore, cocaine use was associated with higher odds of carotid plaque at baseline, suggesting that it is a risk factor for stroke [62].

Genetic variants are linked to increased risk for stroke in cocaine users. However, their precise impact on stroke risk remains unknown, as the association of the identified variants is considered relatively weak [66]. One of these genetic factors is the histone deacetylase 9 (*HDAC9*) gene, which has been associated with large vessel stroke [66,67]. *HDAC9* belongs to the family of epigenetic molecules, known as the histone deacetylases (HDACs), which are involved in the regulation of maladaptive behavioral changes induced by cocaine use [68,69]. There is evidence that overexpression of *HDAC4* (another member of this family) in the nucleus accumbens of the brain can modulate cocaine reward [69]. Moreover, the single nucleotide polymorphism (SNP) rs3791398 on *HDAC4* is associated with carotid intima–media thickness [70]. Based on the above, it is possible that carriers of particular variants on HDACs are more prone to cocaine abuse and have an inherent susceptibility for stroke.

Among 584,115 patients with stroke, identified from the data of the National Inpatient Sample of the Healthcare Cost and Utilization Project, in-hospital outcomes, mortality and comorbidities between patients with stroke following cocaine use and patients with stroke without cocaine use were compared. The results showed that in the users group, cardiovascular incidences were higher than in the non-users group, including valvular disorders (13.2% versus 9.7%, *p* < 0.001), venous thromboembolism (3.5% versus 2.6%, *p* < 0.03), vasculitis (0.9% versus 0.4%, *p* < 0.003), and sudden cardiac death (0.4% versus 0.2%, *p* < 0.02). In the users group, the incidence of epilepsy and major depression was also higher. In the non-users group, the incidence of certain risk factors for stroke (atherosclerosis, elevated cholesterol, hypertension, cardiac circulatory anomalies, diabetes, family history of stroke, paralysis, transient ischemic attack, coagulopathy, deficiency anemia, and disorders of fluid and electrolytes) was higher. Users also presented higher in-hospital mortality, while venous thromboembolism or vasospasm seemed to be connected to cocaine administration. The chronic use of cocaine seems to make users more vulnerable to stroke, but further research is necessary in order to assess cocaine-induced stroke [71].

The frequency and the route of administration seem to play an important role, when assessing the link between cocaine use and the risk of stroke. Following the acute use of cocaine, a 6.4-fold higher incidence of stroke within 24 h for users is reported, compared to those who had never used cocaine. Furthermore, acute cocaine use has also proven more detrimental compared to chronic use. In addition, smoking cocaine presents the highest risk for stroke. In 26 patients, suffering from stroke following acute cocaine use, the prominent route of administration was smoking (“crack”), while in all cases, typical risk factors for stroke, such as hypertension, myocardial infarction, hyperlipidemia, diabetes mellitus, and tobacco use, co-existed. Some patients were multidrug users (heroin and marijuana) [72]. It is rather likely that stroke can occur following cocaine use, even without other risk factors [73].

Neurovascular implications are rather common among cocaine abusers. Among 96 active or former cocaine users, 45 cases of ischemic stroke/TIA were reported, while intracerebral hemorrhage (ICH) and SAH occurred with a similar prevalence of approximately 25%. ICH and SAH were associated with active cocaine use, while ischemic stroke/TIA was more likely to occur in former cocaine users. Regarding the different forms of cocaine, crack is implicated equally in both types of strokes, while cocaine is implicated more in hemorrhagic stroke [59]. In a paper published by Martin-Schild et al., the authors compared the location, demographics, and outcome of patients with ICH. Out of 3241 patients with stroke, 132 (4.1%) were cocaine users, according to the urine drug screen, and 45 had ICH. Six of the 45 cocaine users with ICH were also using other illicit drugs (such as marijuana and amphetamines). The control group consisted of 105 non-users with ICH. The study showed that cocaine users with ICH had a male predominance and were less likely to be Hispanic (11% vs. 28%; *p* = 0.022) and more likely to be African-American (69% vs. 44%). Cocaine users had a higher median diastolic blood pressure (121 (100–126) vs. 110 (107–141)); *p* = 0.024). Furthermore, cocaine users had more severe ICH, compared to the control group. In addition, cocaine use seems to correlate with the emergence of intraventricular hemorrhage (IVH). This study also showed that cocaine users are more likely to die during their hospitalization, compared to the control group [74]. One study investigated the outcome of strokes related to cocaine abuse, compared with strokes that are not related to cocaine. They concluded that younger age and cardiac arrhythmias are associated with cocaine-related strokes. Regarding other traditional cerebrovascular risk factors, no differences were found between cocaine and non-cocaine related strokes [55].

There are several case reports on hemorrhagic or ischemic stroke after cocaine use and the most recent and interesting are presented in Table 2. 

### 2.3. Cannabis

Cannabis is extracted from the plant *Cannabis sativa* and its varieties, *Cannabis Americana* and *Cannabis Indica*, and has two principal preparations, marijuana and hashish, which can be smoked, ingested or inhaled. Delta 9-tetrahydrocannabinol (THC) is the psychoactive cannabinoid in cannabis. Based on the THC content, potency varies in the preparations of cannabis and it is usually higher in hashish than in marijuana [81]. Cannabis substitutes anandamide, a neurotransmitter involved in mechanisms of appetite regulation, memory, reproduction and cell proliferation (the basis of tumor development). 

#### 2.3.1. The Mechanism of Action of Cannabis

The mechanism of action of THC has also been controversial. At first, it was thought that, due to the lipophilic nature, it causes the disruption of the membranes of the cell components. In the 1990s, researchers discovered cannabinoid receptors located in the brain and in the cells of the body, responsible for many of the effects of THC [82]. The molecular mechanism was initially considered nonspecific, of an anesthetic type, for which the lack of stereospecificity of the activity of delta-9-THC and also its lipophilicity was advocated [82]. The first evidence for the specific action of cannabinoids was brought by Howlett, who showed that delta-9-THC inhibits adenylate-cyclase activity in N18TG2 neuroblastoma cells cultured in vitro, and the use of a radiolabeled analogue allowed the detection of cannabinoid sites, specific in the brain [82,83].

There are two types of cannabinoid receptors (CB): CB_1_ in the central nervous system and CB_2_ in the immune system cells [82,84]. High densities of cannabinoid receptors are found in the frontal cortex, basal ganglia, cerebellum, and hippocampus. They are absent in the brain nuclei. The stimulation of these receptors causes the release of neurotransmitters [82]. The main effects of cannabis are relaxation, euphoria and increased self-confidence. Its side effects include cardiovascular complications, peripheral events (such as kidney infarction or peripheral arteritis) and neurological complications [82,84].

#### 2.3.2. The Influence of Cannabis on Stroke

Cannabis causes transient cerebral ischemic attacks (TIAs) and ischemic strokes.

The possible mechanisms through which cannabis can induce stroke include cerebral vasoconstriction, hypotension, vasospasm, impaired cerebral vasomotor function and fluctuations in blood pressure [81,85]. It is possible that all the above could be attributed to the potential of cannabis to induce sympathetic stimulation and decrease parasympathetic activity [8,86]. There is currently increasing scientific interest towards the determination of the dose and duration of cannabis abuse that would lead to a stroke. In a study conducted on the National Inpatient Sample database from USA, a significant increase in symptomatic cerebral vasospasm was observed in marijuana users [87]. In a case of basal ganglia hemorrhage, reported after an increased intake of cannabis, the proposed mechanism for the pathogenesis of intracerebral hemorrhage was the capacity of cannabis to impair autoregulation and to induce transient arterial hypertension [88,89].

**Figure 3:**
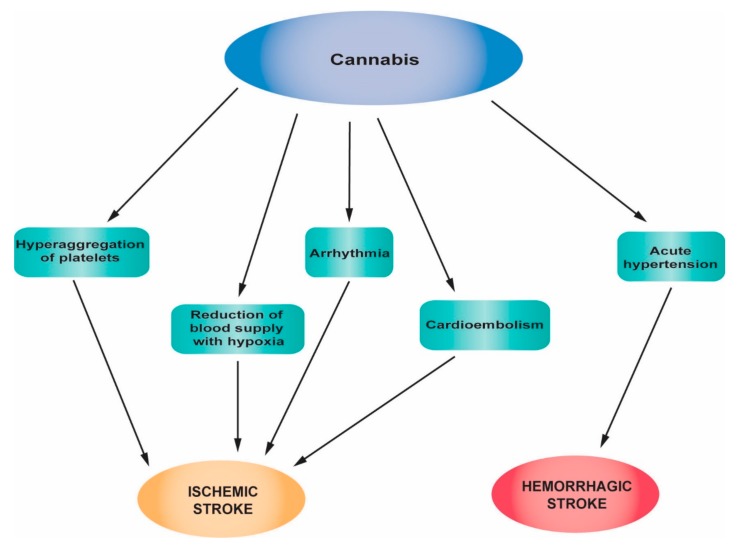
Main pathophysiological mechanisms of stroke associated with cannabis abuse.

Regarding the mechanism by which cannabis induces thrombotic events, one should consider the fact that platelets synthesize endogenous cannabinoids, mainly the Δ 9-tetrahydrocannabinol (THC) metabolite [90]. Via CB1 and CB2 receptors, the platelet membranes are targets for exogenous cannabinoids, resulting in aggregation, which is nonreversible, at high cannabinoid levels [90,91]. Moreover, cannabinoids lead to the increased reactivation of factor VII and elevated ADP-induced aggregation in platelet-rich plasma [91]. An additional procoagulatory effect appears to be the elevated expression of glycoprotein IIb-IIIa and P selectin on the surfaces of the platelets by THC, dependent though by concentration [90]. The stimulation of the sympathetic system and the inhibition of the parasympathetic system, and the inflammatory processes at the level of at the arterial wall, have been described as other possible THC mechanisms of action, resulting to thrombus formation and endothelial erosion at both cerebral and coronary arteries [92,93,94]. Finally, cannabinoids can also lead to the activation, adhesion and aggregation of platelets, as a result of the decreased availability of nitric oxide, due to oxidative stress (which is induced by cannabinoids [90,91]. Figure 3 summarizes the main pathophysiological mechanisms of stroke associated with cannabis abuse.

#### 2.3.3. Clinical Studies, Case Reports and Epidemiology of Stroke Related to Cannabis

The most widely used psychoactive substance in the world is cannabis, with almost 180 million annual consumers [8]. Most users believe that cannabis is a safe recreational drug. Furthermore, because of its therapeutic applications, 15 states of the US have approved it for medical use [81]. In Europe, countries such as Cyprus, Finland, Germany, Greece, Italy, Israel, Norway, Netherlands, Croatia, Czech Republic, Denmark, Georgia, Luxembourg, Malta, Poland, Portugal, San Marino, Switzerland, United Kingdom have already legalized the use of cannabis for medical purposes and other countries are in the process of legalizing it [95].

Following the chronic use of cannabis, psychological and physical dependence are encountered and the withdrawal syndrome includes sleep difficulties and anxiety [81].

In recent years, there have been several case reports, case series and studies that show a link between cerebrovascular events and cannabis use [96,97,98]. It seems that cannabis users are more likely to present with neurological conditions, such as multifocal intracranial arterial stenosis, reversible cerebral vasoconstriction syndrome and chronic use of cannabis can lead to increased cerebrovascular resistance. There seems to be a link between cannabis use and stroke/TIA (odds ratio, 2.30; 95% confidence intervals, 1.08–5.08) [96].

In 48 patients (under 45 years of age) admitted to hospital for ischemic stroke, cardiovascular investigations, blood tests and urine screens for cannabinoids were performed, in order to study stroke in young adults. Urine tests were positive for cannabis in 13 patients. Out of these 13 patients, 21% had a distinctive form of multifocal intracranial stenosis (MIS) and suffered from a severe headache. In seven patients, ischemic stroke was located in the vertebrobasilar territory; in nine patients, MIS was in the posterior cerebral arteries; and in seven patients, it was located in the superior cerebellar arteries. The link between MIS and cannabis was statistically significant (odds ratio, 113 (9–5047); *p* < 0.001) [99].

Studies showed that cannabis can be related to stroke, especially in smokers. Interestingly, in a large cohort of 49,321 Swedish men, born between 1949 and 1951, who had been in the military service between 1969 and 1970, alcohol consumption, cannabis use or tobacco smoking and their association with stroke were studied. Among men who have had a stroke before 60 years of age, the risk factors were often common and included a family history of cardiovascular disease, obesity, high alcohol consumption, and tobacco smoking. Cannabis use was associated with elevated blood pressure and was more prevalent in stroke at a younger age (< 45 years), but cannabis use alone was not reported as a risk factor for stroke in individuals younger than 45 years old [8]. Rumalla et al., conducted a study on patients between 15 and 54 years old, with a primary diagnosis of acute ischemic stroke (AIS). Data were obtained from the Nationwide Inpatient Sample, the largest inpatient database in the US. The purpose of this study was to evaluate the correlation between marijuana use and hospitalization for AIS. The researchers identified an increased incidence of AIS in the marijuana cohort, especially in young patients, who were African American males. Multivariable analysis was applied to investigate the risk factor for the occurrence of AIS involving marijuana use alone or in combination with other risk factors. The analysis showed that marijuana use represented a significant risk factor for AIS hospitalization [87].

Cannabis use has been mainly associated with ischemic stroke. Nevertheless, more recent studies show an association between cannabis use and hemorrhagic stroke. Several recent case reports on cannabis-associated stroke are presented in Table 3. A very interesting case is that reported by Atchaneeyasakul et al., It refers to a 27-year-old man who presented right basal ganglia intracerebral hemorrhage (ICH) following the ingestion of cannabis. No vascular abnormality was observed on digital subtraction angiography (DSA) of the cerebral vasculature, CT angiography of the head, and magnetic resonance imaging (MRI) of the brain. The toxicological tests were positive for cannabinoids, with a serum level of 9-carboxy tetrahydrocannabinol of 222 ng/mL. The patient was not diagnosed with secondary hypertension. The fact that no other risk factor for the basal ganglia hemorrhage was identified in this case supports the role of cannabis in the risk of stroke with robustness [88].

### 2.4. Synthetic Cannabinoids

Synthetic cannabinoids are a new class of psychoactive chemicals, similar in pharmacological action with THC, the active component of *Cannabis sativa* [105]. Synthetic cannabinoids are not derived from cannabis. They are synthetized in the laboratory and they manifest a full agonist activity on cannabinoid receptors, in contrast to THC which is only a partial agonist [105]. They are metabolized to active metabolites that give them a higher potency compared to THC [106]. Although they are labeled “not for human consumption”, they are available in the market as herbal mixtures sprayed with synthetic cannabinoids, in street language known as “spice”, “K2”, “herbal incense”. They are used for recreational purposes and they are called “legal drugs” [106]. Their use has increased in the last years, along with concerns regarding their safety. The market of synthetic cannabinoids is growing very fast and a new compound is synthetized as soon as the previous one is classified as illegal by legislation.

#### 2.4.1. The Mechanism of Action of the Synthetic Cannabinoids

Synthetic cannabinoids act as CB1 and CB2 cannabinoid receptor agonists, similar to tetrahydrocannabinol (THC) but they have a different chemical structure [107]. They cause agitation, anxiety, paranoia, hypertension, rarely myocardial infarction or renal failure [107].

#### 2.4.2. The Influence of Synthetic Cannabinoids on Stroke

Synthetic cannabinoids have been associated with ischemic stroke through various case reports. Unfortunately, epidemiological studies are hard to conduct because these substances are not detected in routine toxicological screen tests [106]. Their increased potency on cannabinoid receptors, their active metabolites, and their cross-reactivity with other receptors induce a strong prothrombotic state, which, in combination with other minor risk factors for stroke, can lead to ischemic stroke [108]. Two case reports that associate AIS with synthetic cannabinoids, support the embolic etiology of stroke which is in agreement with previous reports on severe adverse cardiac events following spice use [109]. In some cases of AIS attributed to synthetic cannabinoid use, past use of cannabis was also reported. In these cases, one could question whether acute synthetic cannabinoid overdose is the actual cause of stroke, or whether chronic cannabis use is also implicated. This theory could be supported by the similarity in the structure of THC and synthetic cannabinoids, which could lead to the same mechanism of cardiovascular injury [106]. Further studies are needed to elucidate the exact mechanism.

The reported cases of hemorrhagic strokes following acute use of synthetic cannabinoids can be explained by the transient vasospasm observed immediately after use [110]. The capacity of synthetic cannabinoids to alter neurotransmitter release from nerve terminals can lead to activation of smooth muscle cells which are associated with disruption of endothelial cell function and can, therefore, lead to ischemia or hemorrhage [111].

**Figure 4:**
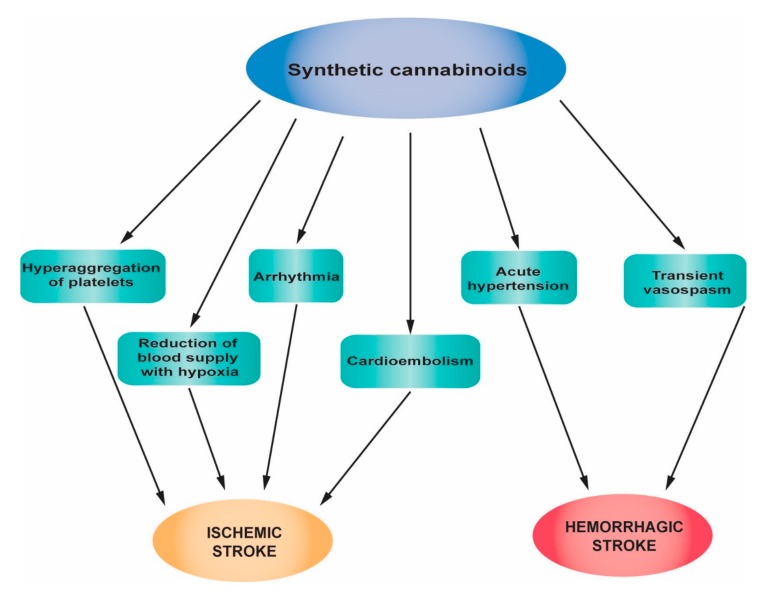
Main pathophysiological mechanisms of strokes associated with synthetic cannabinoid abuse.

Figure 4 summarizes the main pathophysiological mechanisms of strokes associated with synthetic cannabinoid abuse.

#### 2.4.3. Clinical Studies, Case Reports and Epidemiology of Stroke Related to Synthetic Cannabinoid Abuse

To date, only a few studies have investigated the toxic effects of synthetic cannabinoids. Case reports correlate their use with severe adverse and toxic effects, different from those observed after marijuana use. Even deaths have been reported [112].

The association of synthetic cannabinoid consumption and ischemic stroke/TIA was first reported by Bernson-Leung et al.. The group has published two cases of ischemic stroke in young people, pathologies that occurred within hours after a first-time exposure to synthetic cannabinoids. One patient was a 22-year-old woman who developed right middle cerebral artery AIS a few hours after smoking “K2” and the other patient was a 26-year-old woman who developed middle cerebral artery territory infarction after smoking “Peak Extreme”. In both cases, the tests for serum vascular risk factors and hypercoagulability were negative. Both cases presented other minor risk factors for stroke: The 22-year-old woman was taking oral contraceptives and the 26-year-old woman had migraine with aura, took oral contraceptives, was an active smoker and had a family history of superficial thrombophlebitis. Even so, both were young and healthy and, most importantly, AIS occurred within a few hours after the first use of synthetic cannabinoids [113]. Another study reported two cases of middle cerebral artery location of AIS in a 26-year-old man and a 19-year-old woman immediately after smoking “spice”. Both had positive urine tests for cannabinoids and they confirmed the use of synthetic cannabinoids in the past but not in the days that preceded stroke. The synthetic cannabinoid JWH-018 was found in their urine [109]. Faroqui et al. described a case of a 36-year-old African American man, without a medical history with risk factors for stroke, who showed extensive left cervical and intracranial internal carotid artery occlusion AIS after smoking “K2”. He also reported smoking marijuana in the past, but not recently [114].

Only recently, there have been reports linking synthetic cannabinoids to hemorrhagic stroke. Rose et al. reported two cases of SAH after smoking “spice”: A 31-year-old man, for whom the consumption of XLR-11((1-(5-fluoropentyl)-1H-indol-3-yl) (2,2,3,3-tetramethylcyclo-propyl) methanone) was confirmed, and a 25-year-old woman [110]. A summary of the main studies is presented in Table 4.

It is interesting to note that case reports on cannabis-associated stroke increased after the appearance of synthetic cannabinoids on the market. Based on the fact that synthetic cannabinoids are usually used together with cannabis and that they cannot be detected in urine through standard screening tests, further studies are needed to elucidate if cannabis is the real cause of these strokes, or whether a synergistic effect is caused by the concurrent use of cannabis and synthetic cannabinoids. 

### 2.5. Opiates/Heroin

The most well-known substances belonging to the class of narcotic analgesics are morphine and heroin. Morphine is a natural substance extracted from some poppy species grown in South-East and South-West Asia, Mexico and Colombia [116]. Heroin (diacetylmorphine) is a semi-synthetic opioid drug, obtained by a chemical reaction between morphine and acetic anhydride [117]. Originally conceived as a substitute for morphine, heroin has been used in the past for the amelioration of withdrawal symptoms in alcohol-addicted individuals. Unfortunately, the synthetic drug is extremely addictive, causing both physical and psychological dependence [117].

#### 2.5.1. The Mechanism of Action of Opiates/Heroin

Narcotic analgesics have a direct action on the vasomotor center and augment parasympathetic activity, reduce sympathetic activity and induce histamine release from mast cells [118]. These effects cause bradycardia, stimulating cardiac automatism, triggering atrial ectopic, atrial fibrillation, idioventricular rhythm or malignant ventricular arrhythmias. A complication of intravenous use is deep venous thrombosis, originating in the deep or superficial femoral vein, with the consequent risk of massive pulmonary embolism and stroke [9,119].

Morphine is rapidly absorbed and metabolized in the liver, and the main active metabolite is 6-glucuronide-morphine. It has a 2-fold increased potency compared to morphine. At the cerebral level, this metabolite has a 100-fold increased potency compared to morphine [116]. The metabolite 6-glucuronide-morphine is responsible for the analgesic action. Morphine has a plasma half-life of 2–3 h with rapid hepatic metabolism. Urinary excretion of metabolites can be detected in urine for up to 48 h (for occasional users) and for up to a few days (for chronic users) [120]. Due to the fact that heroin is more lipid soluble than morphine, it has an increased mode of action [121]. 

#### 2.5.2. The Influence of Opiates/Heroin on Stroke

A proposed mechanism for heroin-associated ischemic stroke is cardioembolism.

This can occur secondary to infectious endocarditis (which is common in intravenous users), or due to other adulterants found in drugs [122]. The cardiogenic embolic effect of infectious endocarditis is further reinforced by the direct toxic effect of heroin on cerebral arteries [123]. Furthermore, post-anoxic encephalopathy and global hypoperfusion of the brain, due to heroin-induced hypotension, bradycardia, cardiopulmonary arrest, and hypoxia, can also be a possible mechanism [122,124]. Recent reports indicate that heroin-induced hypereosinophilia could be the cause of heroin-induced cerebral infarction. Heroin is known to induce hypereosinophilia in chronic users. Bolz et al. describe the case of a 29-year-old man who admitted sniffing heroin for seven years. He was diagnosed with heroin-induced hypereosinophilia and presented with multiple cerebral infarctions, without having any other cardiovascular risk factors [125]. The mechanism involved in cerebral ischemia could be associated with focal damage of the endothelium of the endocardium and of both small and larger arteries, determined by eosinophilic-associated proteins. This can be associated with increased blood clotting and local hypercoagulation, determined by components of eosinophilic granule [126]. In a study conducted in 2009, Hamzei Moqaddam et al. report that opioid dependence may be considered as an independent risk factor for stroke. The suggested underlying mechanism was that opioid dependence may increase plasma fibrinogen levels, which are known to represent a risk factor for the development of atherosclerosis in the coronary arteries, as well as in peripheral and cerebral vessels, and may, therefore, lead to heart infarctions or stroke [127].

Hemorrhagic stroke induced by heroin has also been reported. The possible pathogenic mechanisms could be: a) the hemorrhagic transformation of ischemic infarction or a hemorrhage determined by pyogenic arteritis and b) the rupture of a mycotic aneurysm [124,128].

**Figure 5:**
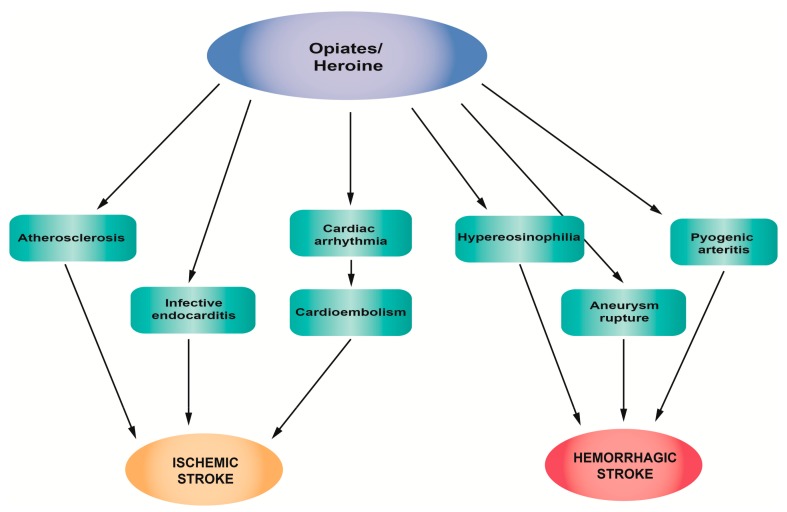
Pathophysiological mechanisms of stroke associated with opiate/heroin abuse.

Figure 5 summarizes the main pathophysiological mechanisms of strokes associated with opiate/heroin abuse.

#### 2.5.3. Clinical Studies, Case Reports and Epidemiology of Stroke Related to Opiates/Heroin Abuse

Heroin-associated stroke has rarely been reported, but intranasal administration can lead to ischemic lesions of globus pallidus [129]. The ischemic pathology of heroin-associated stroke is more common than hemorrhagic forms [130]. Kumar et al. described a case of a 28-year-old woman who admitted using heroin and presented with intraparenchymal hemorrhage in the left frontal lobe without cardioembolic, vasculitic or other etiologies for stroke [128]. In the literature, there are only a couple of other cases of hemorrhagic stroke in young people who use heroin: A 42-year-old man who presented with massive left intracerebral hemorrhage and a 45-year-old man who presented with right basal ganglia hemorrhage [131]. Chronic morphine treatment may be associated with an increased incidence of stroke in patients with malignancies. A higher correlation is encountered in prostate cancer patients, as shown in a 2013 study in Taiwan [132].

Opiate-addicted individuals have a higher risk of stroke than the general population [133]. In Table 5, the most recent case reports that associate narcotic analgesic use and stroke are presented. 

### 2.6. Androgenic Anabolic Steroids

Anabolic androgenic steroids (AASs) are either endogenous (e.g., testosterone) or synthetic, exogenous substances (e.g., nandrolone and stanozolol), acting through specific androgen receptors. AASs are used for the treatment of several disorders, such as hypogonadism, cachexia of various etiologies, hypercalcemia, hypercalciuria, and along with other chronic diseases also in oncology as a supportive treatment [136].

#### 2.6.1. The Mechanism of Action of AASs

The mechanism is complex and is associated with several parameters. More specifically, changes in the lipid profile have also been observed, both at chronic therapeutic doses and during short term treatment, with the reduction in high-density lipoprotein (HDL) cholesterol being the most profound change. Interestingly, molecular biology tests revealed that a concomitant increase in total cholesterol was accompanied by increased mRNA and protein expression of HMG-CoA reductase, a key enzyme in the formation of cholesterol by the liver [137]. The decrease in HDL cholesterol may reach 20% and, similarly, the increase in low-density lipoprotein (LDL) cholesterol may reach 20%, possibly as a result of the lipoproteins’ lipolytic degradation and their subtraction by receptors due to the modification of apolipoprotein A-I and B synthesis [138]. Apolipoprotein B has been connected to atherosclerosis, via the interaction between the arterial wall and LDL cholesterol [139]. Abnormalities in lipoprotein expand the hazard of coronary artery disease by 3–6 fold and it may occur within 9 weeks of AAS use. In addition to its atherogenic effects, the excess of LDL-C may be oxidized at the arterial endothelium leading to impaired endothelium-dependent arterial relaxation via inhibition of nitric oxide production. This could predispose to the development of coronary vasospasm [140]. Fortunately, the effects of lipids appear to be reversible [141].

The effects of anabolic steroids on blood pressure remain conflicting. A few studies have reported elevated blood pressure levels in anabolic steroid users [142], which might be maintained even 5 to 12 months after discontinuation [143]. The mechanism involved could be the ability of AASs to increase the activity of the sympathetic nervous system activity, to baroreflex control and to endothelial dysfunction as well [144]. The mode of action seems to be substance specific. For example, nandrolone has no effects on blood pressure, while the cardiac hypertrophy caused by nandrolone administration was not associated with the systemic renin–angiotensin system but with its effects at a local level. Unfortunately, data so far are not sufficient to settle on whether the prolonged AAS use can lead to irreversible elevated levels of blood pressure [145].

#### 2.6.2. The Influence of AASs on Stroke

Atherothrombosis or embolization could lead to thromboembolic ischemic strokes. Peripheral vascular disease can occur through the same mechanisms. The main action of AASs is anabolism. It is involved in growth-promoting effects on cardiac tissue, following AAS administration and causes hypertrophic cardiomyopathy. Probably as a counteracting effect, apoptotic cell death has also been observed—a process that is mediated by membrane receptor second messenger cascades that increase intracellular Ca^2+^ influx and mobilization, leading to the release of apoptogenic factors [146,147,148]. In vitro studies performed in isolated human myocytes have shown that AASs bind to androgen receptors. Therefore, it is possible that hypertrophy may be induced directly, via tissue upregulation of the renin–angiotensin system [149]. Supporting evidence lies in the fact that the AT1 receptor antagonist prevented similar effects induced by nandrolone administration [150]. Moreover, nandrolone treatment, in combination with swimming training, increased left ventricular angiotensin-converting enzyme (LV-ACE) activity and CYP11B2 expression, implying an elevation in both angiotensin II and aldosterone and the promotion of cardiac dysfunction [151].

Sex hormone-related mechanisms also seem to be involved in the pathogenesis of various cardiovascular disorders, with ischemic stroke included, particularly for men. However, these findings are not specifically informative about endogenous testosterone or testosterone supplementation [152]. Testosterone supplementation for therapeutic purposes has not been conclusively linked with a high thrombotic risk. In a cohort of 3422 male US military service members, aged 40–64 years, treated with testosterone for low testosterone levels, there was no difference in event-free survival with regard to thromboembolism, compared to an appropriately matched control group [153]. On the other hand, elevated testosterone was independently associated with an increased risk for both ischemic stroke (odds ratio 3.9) and cerebral venous thrombosis (odds ratio 5.5) [154]. Nevertheless, the Guidelines of the Endocrine Society suggest that testosterone therapy should be avoided in patients with, among other clinical conditions, elevated hematocrit, myocardial infarction or stroke within the last 6 months or thrombophilia. Furthermore, measuring serum testosterone concentrations and hematocrit is highly recommended [155].

The effect of AASs on the hemostatic system may lead to a prothrombotic profile, depending on the dose and the duration of AAS administration. Low doses decrease platelet threshold activation to collagen. In addition, androgens reduce plasminogen activator inhibitor-1 (PAI-1) levels and increase fibrinolytic activity via high tissue plasminogen activator (t-PA) levels. Both the release of t-PA from endothelial cells into the circulation and the amount of t-PA inhibitor (PAI-1) that is present in the circulation regulate fibrinolytic activity [156,157]. Possible vascular thrombosis due to increased fibrinolytic activity as a result of decreased PAI-1 levels can consequently be speculated [158]. Higher doses have been associated with the elevated aggregation of platelets and possibly affect the activity of vascular cyclooxygenase enzyme, which may lead to a procoagulant state [159]. Several AASs appear to be involved in procoagulatory pathways, by increasing plasma levels of factor VIII and IX [160]. They also increase the aggregation of platelets and the formation of thrombus formation via increased platelet production of thromboxane A2, and via decreased production of prostacyclin and increased fibrinogen levels [139]. At the same time, as animal experiments have shown, extracellular matrix, nitric oxide production and the arachidonic metabolism of endothelial cells and platelets are also influenced [161]. Moreover, both exogenous and endogenous AASs can provoke polycythemia and consequent ischemic cardiovascular events through the reduction of hepcidin and the stimulation of erythropoiesis, by recalibrating the erythropoietin set point [162,163]. Testosterone has also been shown to stabilize telomeres in bone marrow progenitors, which may play a role in increased red cell production [164].

**Figure 6:**
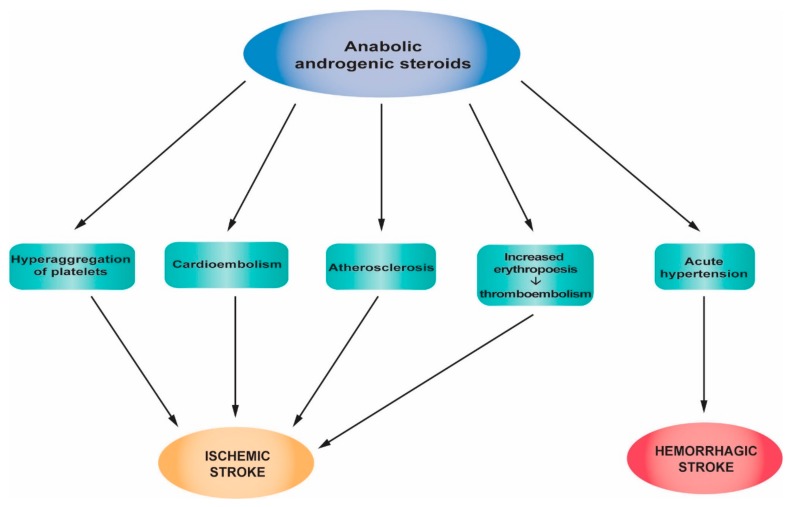
Pathophysiological mechanisms of strokes associated with anabolic androgenic steroid abuse.

Figure 6 summarizes the main pathophysiological mechanisms of stroke associated with anabolic androgenic steroid abuse.

#### 2.6.3. Clinical Studies, Case Reports and Epidemiology of Stroke Related to AAS Abuse

Since the early 1930s, AASs have been extensively used by amateur or professional athletes and the general public for the improvement of physical conditions and athletic performance [165,166,167,168]. When used for ergogenic or recreational purposes, the doses are usually 5–15 times higher than the recommended therapeutic ones [145,167,169]. At such high levels, AASs can cause a number of serious side effects, including liver dysfunction, renal disorders, cardiotoxicity and potentially stroke [136].

Indeed, athletes abusing AASs for years have a high probability to develop atherothrombotic phenomena (cardiovascular and cerebrovascular disorders, such as cerebral ischemia, i.e., transitory ischemic attacks resulting in stroke, peripheral artery occlusive disease and venous thromboembolism) [143]. These phenomena can be attributed to arterial hypertension, lipid metabolism disorders, increased vascular tone and increased platelet counts and hematocrit [139,145,170]. The reversibility of such myocardial and vascular effects after discontinuation is still controversial [171]. Several case reports describe stroke in AAS abusers, and the most interesting ones are summarized in Table 6.

Table 7 summarizes the association between the different classes of drugs of abuse with different types of stroke.

## 3. Management

Stroke can occur either in minutes/hours following illicit drug use or later as a consequence of complications, such as vasculitis or endocarditis, resulting in septic emboli [45,174].

Acute stroke is a medical emergency. Patients should be transported by ambulance to a medical facility that is organized and equipped to manage acute stroke as soon as possible after symptom onset and capable of offering emergency treatments such as intravenous thrombolysis and endovascular thrombectomy—organized acute stroke unit management. These treatments are typically offered in departments of neurology with organized stroke centers [40].

The outcome of ICH depends on the hematoma location and volume, the promptness of treatment, and the management of associated diseases. The mortality of ICH remains very high. For those who survive, recovery is difficult and long lasting, with a negative impact on quality of life. Risk factors, such as high blood pressure, smoking, obesity and drug use, play an important role. Prevention plays a central role and can be favorably influenced by changing lifestyle and taking therapeutic measures, especially for hypertension control.

## 4. Conclusions

Drug abuse represents a major social and public health problem, with huge financial implications. Epidemiological studies and case reports have shown that drug abuse is a risk factor for both hemorrhagic and ischemic stroke. Stimulants, such as amphetamines, amphetamine derivatives, and cocaine have been associated with both types of stroke—more so of the hemorrhagic type. “Crack” cocaine can cause both acute ischemic stroke and hemorrhagic strokes, while cocaine hydrochloride is more likely to cause hemorrhagic strokes. Stroke can emerge after cocaine use, even in the absence of other traditional stroke risk factors. The association between cannabis, synthetic cannabinoids, or opioid/heroin use and stroke has not been entirely proven by epidemiological studies that offer contradictory findings. New case reports describe the correlation between cannabinoids and synthetic cannabinoids and hemorrhagic stroke. Anabolic androgenic steroids are associated with cardiotoxicity and atherothrombotic phenomena which can lead to ischemic stroke. Given the epidemic of illicit drug use, we recommend that every hospitalized stroke patient, and especially those who are young for stroke, is subjected to toxicological screening.

## Figures and Tables

**Table 1:** Characteristic case reports that associate amphetamines and amphetamine derivatives abuse with stroke.

Subject/Age	Substance Exposure	Symptoms	Diagnostic Approach	Diagnosis	Intervention	Evolution	Reference
Female, 23, no previous medical history	Took 4-fluoroamphetamine 4 h before, concomitant use of cannabis 7 h before	Collapsed at a dance event, no neurological deficits, sleepy and headache, decreased consciousness 1.5 h later, weakness of the right arm and leg	Plain computed tomography scan (computed tomography (CT) scan); CT angiography	Intracerebral hemorrhage in the left hemisphere; dilated non-responsive right pupil (false localizing sign)	Acute neurosurgical intervention: Hematoma evacuation, removal of the bone flap due to persistent intraoperative brain swelling	Right-sided hemiparalysis and severe aphasia. Replacement of the autologous bone graft after 4 months without complications. Able to talk in her native language and walk with supportive measures	[44]
Female,23, no medical history	Took 110 mg 4-fluoroamphetamine the night before, concomitant use of four units of alcohol	Severe headache, nausea, followed by vomiting 5 h after the intake, dizziness, photophobia	CT scan	Small subarachnoid hemorrhage at the right frontal side	Discharged after 24 h	Headache for weeks that gradually declined cognitive problems. Inability to work for several months	[44]
Female, 29, progressive headache and diplopia for 2 weeks, no medical history	Intravenous methamphetamine use	A 2-day history of left-sided hemiparesis and dysarthria	Cranial nerve examination, CT brain imaging without contrast medium, magnetic resonance imaging (MRI), angiogram	A 25 × 25 × 20-mm hyperdense lesion within the right cerebellopontine angle Initially thought to represent an extra-axial mass (meningioma), confirmed to be a large brainstem hemorrhage, extended from the inferior midbrain to the pontomedullary junction	Transferred to rehabilitation	Deterioration of left hemiparesis, dysarthria and dysphagia after 1 month. No underlying vascular abnormality observed	[45]
Male, Caucasian, 53, history of head and neck squamous cell carcinoma post-surgery and radiation (13 years before), hypothyroidism, hyperlipidemia, gastrointestinal reflux disease	Treatment for Attention Deficit Hyperactivity Disorder (ADHD) with mixed amphetamine salts, starting 5 mg/day to 15 mg/day over 4 months	Posterior headache with left-face numbness, diplopia 2.5 months after last dosing scheme	Head CT without contrast agent; MRI; transthoracic echocardiogram	Right posterior paramedian midbrain hematoma with cerebral aqueduct effacement and mild ventriculomegaly. No hypertension, arteriovenous malformation, cavernous malformation, or aneurysms	-	-	[46]
Male, 31	Amphetamine abuse	-	Transcranial color-coded Doppler sonography; angiography	Intracerebral hemorrhage, diffuse cerebral vasospasm	Surgical removal of intracerebral hemorrhage, pharmaceutical treatment	-	[47]
Male, African-American, 20	Took 3,4-methylenedioxymeth-amphetamine (MDMA), concomitant use of marijuana and beer	Non-verbal, vomiting and aphasic upon presentation, no sign of trauma, 18 h after ingestion developed right-sided weakness, left-sided facial droop and bilateral hyperreflexia in the lower extremities	MRI; carotid ultrasound; magnetic resonance angiogram of the brain	Left middle cerebral artery complete infarction, no significant stenosis, mild to moderate stenosis observed on the distal left internal carotid artery	Transferred to rehabilitation	-	[48]
Female, 36, history of migraine	Methamphetamine use, concomitant use of oral contraceptives	Sudden onset of speech difficulty and right-sided weakness	Head CT; MRI of the brain; MR angiography	Small infarct in the left frontal lobe, focal narrowing in the left internal carotid artery	Pharmaceutical treatment: IV heparin, discharged on warfarin 5 days after stroke; after 8 months, warfarin was replaced with aspirin 81 mg/day	Recovered after 4 months with only mild expressive aphasia	[49]
Female, 29	History of methamphetamine use for 10 years	Sudden right-sided weakness and speech difficulty 4 days after last use of methamphetamine	Head CT, MRI, MR angiography	Large left middle cerebral artery (MCA) infarct, MCA infarct with hemorrhagic transformation	Discharged after 4 days on aspirin treatment, on day 5th showed worsening deficit, hospitalized; stent-assisted transformation applied	Recovered only with moderate expression aphasia and mild right-hand weakness within 4 months	[49]
Male,31	Methamphetamine ingestion approximately 0.25 and 0.5 g Urine screen positive also for tetrahydrocannabinol (THC)	Severe headache, nausea, vomiting, left-side of the body felt numb, slurred speech, died the next day	Autopsy	Cerebral edema, subarachnoid hemorrhage over the cerebral convexities bilaterally, intracerebral hemorrhage lateral to the basal ganglia extending to involve the lateral aspect of the putamen, external capsule claustrum, insula, and superior longitudinal fasciculus of the right cerebral hemisphere (3.5 cm by 4.5 cm) No evidence of inflammation or vasculitis		Death	[38]
Male, Caucasian, 33, amphetamine addict	Amphetamine and methamphetamine ingestion. Low concentrations of methadone and codeine in the blood	Bilateral cerebral infarction associated with multi-organ failure	CT scan, autopsy	Extensive infarction of both cerebral hemispheres; symmetrical necrosis of the white matter of both cerebral hemispheres in the autopsy		Died 19 days after hospital admission	[42]
Female, 30, no significant medical history, non-smoker, very light alcohol consumer	Ecstasy ingestion one night before the presentation	Right-sided weakness, global aphasia, right neglect, and right hemiparesis	Brain CT scan; ultrasound of the extracranial carotid arteries; transcranial color-coded Doppler (TCCD); MRI	Left parietal hypodensity consistent with left middle cerebral artery (MCA) infarction; irregularity of the left MCA	Aspirin 100 mg/day	TCCD studies showed normal velocities in the MCA 3 months after onset	[33]
Female, 19, duodenal ulcer at 16, no other medical history, no family history of stroke	Methamphetamine intravenously four times over 2 months, wash-out for 3 months, concomitant use of cigarettes and alcohol	Severe right-sided headache, blurred vision on the left side and numbness of the left arm and leg upon admission, severe headache every time associated with use	Brain CT, MRI and magnetic resonance angiography	Right occipital infarction, segmental narrowing of the right posterior cerebral artery with characteristics of vasculitis	Discharged one week after admission	The right occipital infarction faded with mild atrophy, left superior quadrant hemianopia remained and had persistent headaches 4 months later	[32]

**Table 2:** Characteristic case reports studies that associate cocaine abuse and stroke.

Subject/Age	Substance Exposure	Symptoms	Diagnostic Approach	Diagnosis	Intervention	Evolution	Reference
Male, African American, 65, diabetes, heart diseases, hepatitis C	Smoking crack cocaine before symptom onset, admitted to intermittent cocaine abuse	Left arm pain described as feeling like “jumping out of the window”	Head CT scan; carotid ultrasound; CT angiography of head and neck	Acute 2.2-cm intraparenchymal hemorrhage that presented in the posterior right parietal lobe vasogenic edema	Send to the rehabilitation unit	Left arm pain resolved after 24 h	[75]
Female, African-American, 66, multi-substance abuser, hepatitis C, heart diseases	Urine samples positive for cocaine	Somnolent a day prior to admission, confused in the day of admission, short-term memory loss, unable to perform usual daily activities	Brain CT; CT angiogram of the head and neck; MRI of the brain associated with MR venogram	Infarction in bilateral posterior inferior cerebellar artery and hippocamp showing multifocal punctate infarcts in the basal ganglia and bilateral posterior cerebral artery secondary to severe vasoconstriction	Neurosurgery consult for possible external ventricular drain placement and posterior fossa decompression	Mental status improved during hospitalization; discharged to a rehabilitation center after 7 days with persistent problems of memory and inability to recognize faces	[76]
Male,22, hypertension and cocaine abuser	Positive for cocaine and tetrahydrocannabinol	Right hemiplegia associated with motor and sensitive aphasia	CT scan	The ischemic region in the left medial cerebral artery region with increased cerebral edema and cerebral midline displacement of 9 mm on the subfalcine region	Not suitable for surgery due to complications	Died in the hospital	[77]
Female, 39, smoker, no other risk factors for stroke	Urine screening positive for cocaine	Global aphasia, left-side total gaze paresis, 7th cranial nerve right-side partial paresis and right hemiplegia	Non-contrast brain CT	Left ischemic stroke—hyperdensity in the left middle cerebral artery (MCA); occlusion in the left and right MCA and an irregular profile of the left internal carotid artery (ICA)	Endovascular treatment, intra-arterial administration of 40 mg of recombinant tissue plasminogen activator (rtPA) associated with a self-expandable and retrievable stent	After 3 months from the event, ischemia at the left basal ganglia	[78]
Male, 31, no medical history	Positive urine screening for cocaine and negative for other drugs	Found unresponsive 6 h after excessive alcohol and intranasal cocaine abuse	MRI; intra- and extracranial CT angiography	Globus pallidus and the vascular watershed zones presents acute bilateral ischemia	-	Consciousness improved progressively; clinical improvements, but mental slowing, executive dysfunction, hypophonia, and verbal fluency deficit persisted	[79]
Female, 31, no medical history, occasional alcohol consumer and smoker	First time snorted cocaine hydrochloride associated with 500 mL of vodka	Acute onset of right hemiplegia and left hemiparesis evolving into quadriplegia	MRI	Thickened pons with focus localized in his central part on the left side (20 mm) (ischemic change)	After 17 days of hospitalization, transferred to rehabilitation	The movements of the left side of the body improved slowly and the rehabilitation continues in ambulatory	[80]

**Table 3:** Characteristic case reports that associate cannabis abuse with stroke.

Subject/Age	Substance Exposure	Symptoms	Diagnostic Approach	Diagnosis	Intervention	Evolution	Reference
Female, 51, asthma	Long-term cannabis user, positive urine screening for cannabis, a large amount of cannabis was consumed prior to the onset of symptoms	Left-side upper and lower extremities weakness	Head CT scan	Acute right cerebral infarct; after 30 min from arrival, developed in the left pons new hemorrhage associated with decompression on the lateral and left ventricles	Pharmaceutical treatment: Labetalol, recombinant tissue plasminogen activator	Died	[100]
Male, 27, without any known medical history	Single raw cannabis consumption (confirmed by a blood test) just before symptom onset	Sudden progressive left-sided weakness, degradation in mentation, nausea, and vomiting	Brain CT without contrast media; CT angiography; MRI of the brain	Right basal ganglia ICH measuring 32 × 24 mm with extension into the ventricles with mild hydrocephalus, no vasculature abnormality	Intubation and placement of an external ventricular drain, treatment on recombinant tissue plasminogen activator	Improvement of motor function, left hemiparesis	[88]
Female, 14, no remarkable medical history	Toxicological screening positive for cannabis 2-year history of daily cannabis use	Generalized tonic–clonic seizures	Head CT, electroencephalography (EEG); MRI	Multiple ischemic infarcts located in basal ganglia, left frontal lobe, and genu of corpus callosum, which had both chronic and acute features	After stabilization, transferred to rehabilitation	Complained of chronic headache, learning disabilities	[101]
Male, 25	Cannabis ingestion one night before Concomitant ingestion of alcohol	Drowsy, talking irrelevantly and the state degraded	Non-contrast CT of the brain; Coronary CT angiogram	Acute infarct in the right frontoparietal region	After hospitalization was discharged in a stable condition	Left-sided weakness improved	[102]
Male	Marijuana History of smoking marijuana from the age of 1	Presented with weakness of leg, arm and face associated with slurred speech 90 min after smoking marijuana Recurrence of the symptoms twice	Brain CT scan, CT angiogram and MRI	Right lentiform nucleus presents subtle hypodensity; no evidence of vasospasm, thrombus or dissection	Heparin treatment after a recurrent episode of focal neurological deficits	After 2 months, he presented residual weakness in the left arm and leg, left facial droop and spastic tone	[103]
Male, 33, smoker	Urine toxicologic screening positive for cannabis Heavy user of cannabis for 15 years	Transient left hemiparesis and dysarthria, no altered consciousness, chest pain one day before	Brain MRI and CT angiography	The presence of multi focal acute infarctions in the bilateral watershed zones between middle and anterior cerebral artery territories and the right middle cerebral artery territory. Cardioembolic stroke produced by acute myocardial infarction (likely related to cannabis use)	-	No recurrence in the following 6 months of cardiac or neurologic symptoms	[104]
Male, Caucasian French, 24, no medical history	Urine toxicology positive for cannabis; heavy cannabis use one night before admission Regular cannabis smoker for four years	Non-reactive state, with seizures	Cerebral CT scan, EEG, MRI, Doppler examination, magnetic resonance angiography, and angiography	Infarcts in the insular mantle and the lenticular and caudate nuclear structures exclude all other causes of stroke in young people	Treated in the hospital until recovery and transferred to the psychiatric department to be treated for behavioral disorders	In the following 1 and a half years, he returned on seven occasions for generalized tonic–clonic seizures	[98]
Male, 36, with no history of migraine or other known vascular risk factors	Urine toxicological screening positive for cannabis Heavy hashish consumption and alcohol before the symptoms Sporadically hashish user	An acute episode of isolated aphasia, followed by convulsive seizures	Cranial MRI and MR angiography	Had 2 acute ischemic infarcts, one on the left temporal lobe and another area of silent ischemia in the right parietal lobe	Treatment with ticlopidine	After 1 year, a new episode of aphasia and right hemiparesis immediately after hashish smoking and a new episode after 1 and a half years again after hashish use Between the two episodes, he denied consumption	[93]

**Table 4:** Characteristic case reports that associate synthetic cannabinoid use with stroke.

Subject/Age	Substance Exposure	Symptoms	Diagnostic Approach	Diagnosis	Intervention	Evolution	Reference
Male, African American, 36, no history of stroke or coagulopathy or blood disorders	Reported taking K2 on the night before symptom onsetConcomitant use of marijuana in the past	Had a 1-day history of aphasia and weakness in the right side of the body	Non-contrast CT of the head; computed tomography angiography (CTA); MRI; MR angiography	A thrombotic event that lead to an acute ischemic infarct with left MCA distribution characterized by hypodensity in the left basal ganglia and a left hyperdense MCA; a large filling defect observed from the origin of the left ICA into the intracranial portions of the ICA	Aspirin, clopidogrel and enoxaparin	After 10 days, the patient was discharged for short-term rehabilitation after gradual improvement	[114]
Female, 22, in treatment with atomoxetine and estrogen-containing oral contraceptive	Smoked K2; concomitant use of THC, benzodiazepine and salicylates as they were positive at urine toxicological test	While smoking K2 presented dyspnea, palpitations and angor animi. Few hours later after smoking K2, developed dysarthria and difficulty standing	Head CT, MRI, and CT angiogram	Right middle cerebral artery AIS; proximal right M1 occlusion with distal reconstruction	Aspirin	In follow-up, presented limited ambulation and no use of her spastic left arm	[113]
Female, 26, smoker, used estrogen-containing oral contraceptive, suffering from migraine with aura	Smoked ‘Peak Extreme’	The next morning after smoking drugs, presented with felt-sided numbness, left facial weakness and dysfluency	CT angiogram, MRI, and head CT	Near occlusion of the right M1 segment with extensive infarction in the middle cerebral artery territory	Warfarin	Improved speech and comprehension	[113]
Male, 33, no medical history	Smoked two “joints” of synthetic cannabinoid product 10 min prior to the onset of symptoms; urine positive also for opiates; synthetic cannabinoid XLR-11-1-(5-fl uoropentyl)-1H-indol-3-yl) (2,2,3,3-tetramethylcyclopropyl) methanone was confirmed in the product used	Right-sided weakness and aphasia	Non-contrast head CT, and electrocardiography	Acute infarction located in the left insular cortex	Aspirin	The neurological problems were completely resolved in 3 days in the hospital; no return to follow-up	[115]
Male, 26, no family history of any stroke risk factors, non-smoker, non-alcohol consumer	Smoked spice “a few hours prior” to his symptom onset; concomitant use of marijuana in the past but not recent	Weakness of right side of face and arm, dysarthria, expressive aphasia that occur suddenly	Non-contrast head CT; CT perfusion; CT angiography; MRI	Hyperdense left middle cerebral artery (MCA); a large area of penumbra without core infarction; left MCA clot	Received IV tissue plasminogen activator (t-PA)	Improved clinically and did not return to follow-up	[109]
Female, 19, smoker, anxiety disorder and panic attacks	Smoked spice; urine drug screening positive for cannabinoids and confirmed for JWH-018	A few minutes after smoking spice, the patient lost consciousness and started vomiting; mental status was persistently altered for several hours; presented with “shaking movements” of the legs and arms according to witnesses	CT angiogram and MRI	Infarctions in the left MCA with large distribution associated with punctate infarcts localized in the right cerebral hemisphere	-	She stabilized neurologically, but right hemiparesis and expressive aphasia remained at a follow-up office visit	[109]
Male, 31	Smoked spice; toxicological tests confirmed XLR-11	Generalized seizure	Head CT and digital subtraction angiography (DSA)	Hemorrhage in the bifrontal subarachnoid associated with left frontal and right parieto-occipital intraparenchymal hemorrhage	Intra-arterial verapamil	After 10 days from the event the paralysis of left leg, left homonymous hemianopsia and mentation improved	[110]
Female, 25, preeclampsia	Smoked synthetic marijuana; concomitant use of marijuana	Seizure after smoking synthetic and nonsynthetic marijuana; left leg monoplegia	CT, MRI, and DSA	SAH in the bilateral Sylvian fissures and interpeduncular and prepontine cisterns; restricted diffusion localized in the right frontal lobe, left cerebellum, left temporal lobe and bilateral parietal and occipital lobes, which is consistent with the diagnosis of multifocal AIS	Intra-arterial verapamil	Follow-up DSA showed worsening vertebrobasilar vasospasm	[110]

**Table 5:** Case reports that associate narcotic analgesic use with stroke.

Subject/Age	Substance Exposure	Symptoms	Diagnostic Approach	Diagnosis	Intervention	Evolution	Reference
Female, 28	Admitted to using heroin	Altered mental status	Head CT	A large 5.1 × 5-cm intraparenchymal hemorrhage in the left frontal lobe, vasogenic edema, and a 5-mm midline shift	Surgical intervention was unnecessary. After discharge, was transferred to rehabilitation	Improvement in cognitive function was mild; the patient continue to be confused and presented significant memory loss	[128]
Male, 29, without cardiovascular risk factors	Sniffed heroin with regularity in the last seven years	Left-sided hemihypesthesia and gait disturbance	MRI and MR angiography	Multiple cerebral and cerebellar areas of diffusion restriction in different territories; heroin-induced eosinophilia	Steroid pulse treatment (methylprednisolone 250 mg IV) in the first three days followed by another 21 days of oral prednisolone (60 mg)—for eosinophilia and antiplatelet therapy with aspirin	A slight improvement in his sensorium and gait but only incomplete recovery	[125]
Male, 33	Heroin inhalation	Amnesia 48 h after first heroin inhalation	MRI	Cortical laminar necrosis of the left hippocampus without vascular abnormality	-	Impaired performance on the verbal and visual level	[134]
Male, 33	Used heroin for 13 years Concomitant use of methamphetamine. For 6 months, started methadone treatment to quit heroin	Found unconsciousness	Brain CT and MRI	Acute ischemic strokes localized in bilateral fronto-parieto-temporal white matter and in bilateral corona radiate. Damage was noted in the bilateral globus pallidus and left cerebral peduncle; rhabdomyolysis	Active treatment in the intensive care unit	-	[135]

**Table 6:** Case reports that associate androgenic anabolic steroid (AAS) abuse with stroke.

Subject/Age	Substance Exposure	Symptoms	Diagnostic Approach	Diagnosis	Reference
Male, 27, with an American father and a mother who was half Japanese, no known stroke risk factors, regularly training, AAS user	Methasterone, prostanozol for the past 6 months	Sudden right hemiparalysis, homonymous hemianopia, dysarthria, tinnitus, and double vision in the middle of muscle training	MRI with and without gadolinium enhancement, MR angiography, three-dimensional CT angiography, carotid ultrasonography, transcranial Doppler and transesophageal echocardiography, and duplex ultrasonography	Cardiogenic embolism and atrial septal aneurysm and large patent foramen ovule, suspected deep vein thrombosis	[170]
Male, 37, no history of alcohol or any other substance abuse, negative medical and family histories	Methandienone, methenolone acetate for the past 2 years	Acute right-sided hemiparesis (grade 3) with right-sided facial weakness, associated with a confused state followed a first-ever experience of generalized tonic–clonic seizure	Brain CT and MRI, ECG, chest X-ray, abdominal ultrasound, and echocardiography	Chronic infarction in the left frontal lobe and subacute left temporoparietal infarction Dilated cardiomyopathy and multiple thrombi in the left ventricle Hepatomegaly, mild ascites and bilateral pleural effusion in addition to a grade I nephropathy	[140]
Male, 16, healthy bodybuilder (weight 87 kg and height 181 cm), unremarkable past medical record	Concomitant use of cannabis (up to 1.5 g/day) and methandrostenolone (40 mg/day) for the past 5 months	Sudden dizziness and right hemiparesis	Cerebral CT, MRI, conventional and magnetic resonance angiography, transesophageal echocardiography, cervical Doppler duplex ultrasound, transcranial Doppler, and ECG	Acute ischemic stroke	[156]
Male, 39, bodybuilder, 3 months earlier sudden loss of vision in the left eye, weakness and numbness in the left upper and lower limbs, lasting less than 1 h, refused admission to hospital	Intramuscular injections of nandrolone twice weekly for the past three years	Dizziness and expressive aphasia for the last 6 h	Brain CT and MRI, ECG, chest X-ray, echocardiography, and magnetic resonance angiography	Dilated cardiomyopathy with LV thrombus formation; embolic stroke and peripheral vascular disease as a complication of the former	[141]
Male, 31, kickboxer	Nandrolone, testosterone clenbuterol since the age of 16; cocaine, ecstasy and alcohol abuser for three years	Patient disoriented in space, mild dysarthria without aphasic elements, oculocephalic preference to right, left homonymous hemianopsia, paresis (3/5), hemicorporal anesthesia on the left side and somatoagnosia	Cranial CT, cerebral arteriography, transesophageal and transthoracic echocardiography, and magnetic resonance angiography	Acute ischemic stroke: Cerebral infarction due to occlusion of the artery cerebral media of unknown etiology	[172]
Male	Injectable (nandrolone decanoate) and oral (methandrostenolone/danabol) three months prior to the incidence Previous intravenous (heroin), and inhaled (marijuana) drug use	Visual disturbances and left-sided weakness commencing 24 h prior to presentation Homonymous hemianopia, mild left-sided weakness in his upper limbs and ataxia in his left upper limb, and high hemoglobin (200 g/L)	Brain magnetic resonance, magnetic resonance angiography, transthoracic echocardiogram, and 24-h Holter monitoring, extensive hematological screening, and thrombophilia screening	Cerebral infarction: Extensive region of acute infarction in the right posterior cerebral artery territory and ongoing occlusion in his right posterior cerebral artery Polycythemia	[173]

**Table 7:** The incidence of ischemic stroke and hemorrhagic stroke in different classes of drugs of abuse.

Drugs of Abuse		Ischemic Stroke	Hemorrhagic Stroke
Amphetamines		+	+++
Amphetamine derivatives		+	+++ Risk in young people without comorbidities
Cocaine		In those with a history of use	In active users
Cocaine	Hydrochloride	+	+++
	Crack	++	++
Cannabis		++	+ In recent case reports
Synthetic cannabinoids		++	+In recent case reports
Opiates/Heroin		++	+In recent case reports
Anabolic androgenic steroids		++	

+ mild evidence. ++ medium evidence. +++ high evidence.
